# Retained ureteral stents, an avoidable source of morbidity: 10 years´ experience from a single tertiary care centre

**DOI:** 10.11604/pamj.2022.42.68.29935

**Published:** 2022-05-25

**Authors:** Mayank Agrawal, Venkat Arjun Gite, Prakash Sankapal, Mudit Maheshwari, Akash Shah, Sabby Dias, Shashank Sharma

**Affiliations:** 1 Department of Urology, Grant Government Medical College and Sir JJ Hospital, Mumbai, India

**Keywords:** Morbidity, stents, time, ureter

## Abstract

**Introduction:**

ureteral stents are used in managing various urological conditions. When these stents are left indwelling for a prolonged time, it results in complications like stent migration, fragmentation, and encrustation. The aim of this retrospective observational study is to analyse the incidence, risk factors, and morbidity associated with retained ureteral stents.

**Methods:**

the retained/forgotten ureteral stents were defined as the stents with an indwelling period of more than six months. The records of all such patients from January 2010 to January 2020 were retrospectively reviewed. The primary reason for the placement of a stent, total indwelling time, the reason for prolonged indwelling time, and part(s) of the stent encrusted were retrospectively reviewed. Single/multistage endourological procedures were used to make the patients remove the retained stents and stone free. The type, number of procedures, and total number of sessions needed were noted.

**Results:**

data of 114 patients was reviewed retrospectively. Most patients presented with abdominal pain (62 patients, 54.4%), and dysuria (41 patients, 35.1%). An average of 1.7 sessions (range 1-4) were needed to make the patients’ stent and stone free. During these sessions, single/multiple procedures (endoscopic/open/combined) were performed. Nine patients (7.9%) had permanent loss of renal unit function and who needed a nephrectomy. Poor compliance (45.6%), unawareness (35.1%), and misconception that the stent would last a lifetime (12.3%), were the most common reasons for retained ureteral stents. The incidence rate of retained stents fell from 1.1% to 0.5% after the “three steps” prevention check method was in-cooperated to ensure timely follow-up of the patients.

**Conclusion:**

retained ureteral stents are a significant source of morbidity, which is avoidable by ensuring timely removal. Sincere efforts should be made to make patients aware of an indwelling foreign body. Prevention is the best strategy.

## Introduction

The use of ureteral stents was first described in 1967 [1]. They are primarily used for managing ureteral obstruction due to stones, tumours, external compression, fibrosis, and for providing drainage after ureteral surgery or iatrogenic injuries [2]. They should be removed timely [3]. Failure to do so results in retained or “forgotten” ureteral stents. This results in complications in the form of stent encrustation, migration, fracture, stone formation, adjacent organ penetration, urinary tract infections (UTI), ureteral erosion, or fistula formation [4,5]. Many of these patients remain asymptomatic for months, and such forgotten stents are detected incidentally, resulting in late presentation. Patients who are symptomatic present with dysuria, lower urinary tract symptoms, flank pain, or haematuria [3,5]. Radiological investigation in form of non-contrast computed tomography (NCCT) or contrast-enhanced computed tomography (CECT) of the kidney, ureter, and bladder region (KUB) is needed to assess the condition of the retained stent, associated stones, if any, and the anatomy of the affected KUB region [1,5]. The primary concerns associated with retained stents are the need for multiple surgical procedures to remove them and in some cases the irreversible loss of renal function. Endourological procedures are the mainstay of surgical management. However, single or at times multiple sessions might be needed to remove these stents [6]. This retrospective observational study aims to look at the incidence, highlight the risk factors, and describe the morbidity associated with retained ureteral stents. We have also analysed how ensuring a timely follow-up can affect the incidence of retained DJ (double J) stents.

## Methods

**Study design:** this is a retrospective observational study that was conducted at a government-run tertiary care centre in western India. The retained/forgotten ureteral stents were defined as the stents with an indwelling period of more than six months.

**Duration/period of study and study population:** the records of all such patients diagnosed and treated from January 2010 to January 2020 were retrospectively reviewed. This constituted the sample size.

**Inclusion and exclusion criteria:** the study included patients of all age groups and gender. In this study, the patients were either referred from other hospitals or were previously operated on at our centre. Only the stents with a recommended indwelling time of three months were included in the study.

**Ethical consideration:** the institute ethics committee approved the study. (Ethics committee no. IEC/Pharm/RP/362/Mar/2021).

**Variables:** the parameters recorded were the patient´s demographic, medical history, presenting complaints, the primary reason for the placement of a ureteral stent, total indwelling time of the stent, and the reason for prolonged indwelling time. The part(s) of the stent encrusted (upper coil, lower coil, and body) were reviewed Based on the radiological investigations. Additionally, the type, the number of procedures performed, and the total number of sessions (single/multistage) needed to remove the stent and associated stones (if any) were also noted.

**Figure 1:**
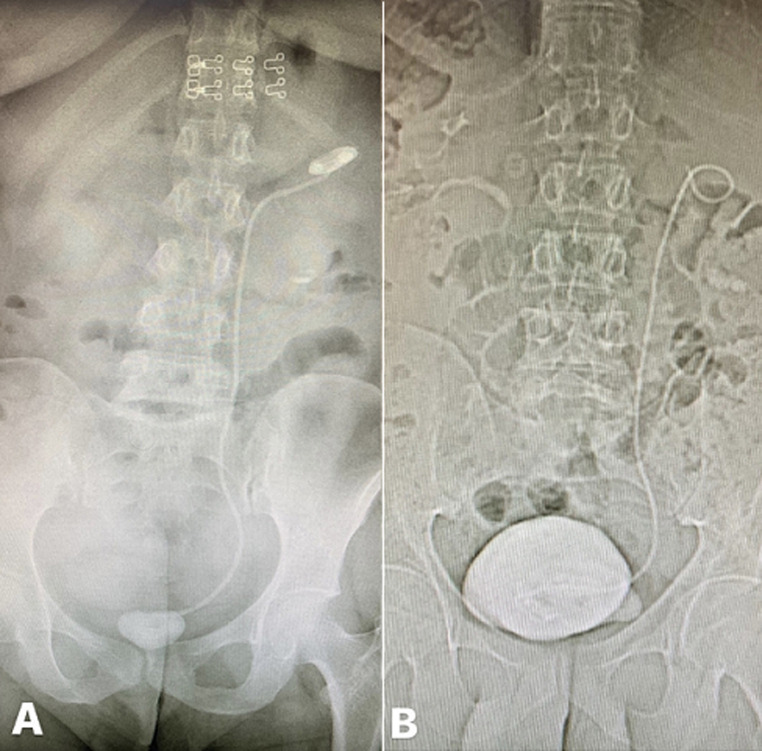
A) plain radiograph showing a retained ureteral stent with encrustation of upper and lower coils; the patient underwent cystolithotrity and percutaneous nephrolithotomy; B) a plain radiograph showing a large bladder stone encompassing the distal coil of a retained ureteral stent

**Figure 2:**
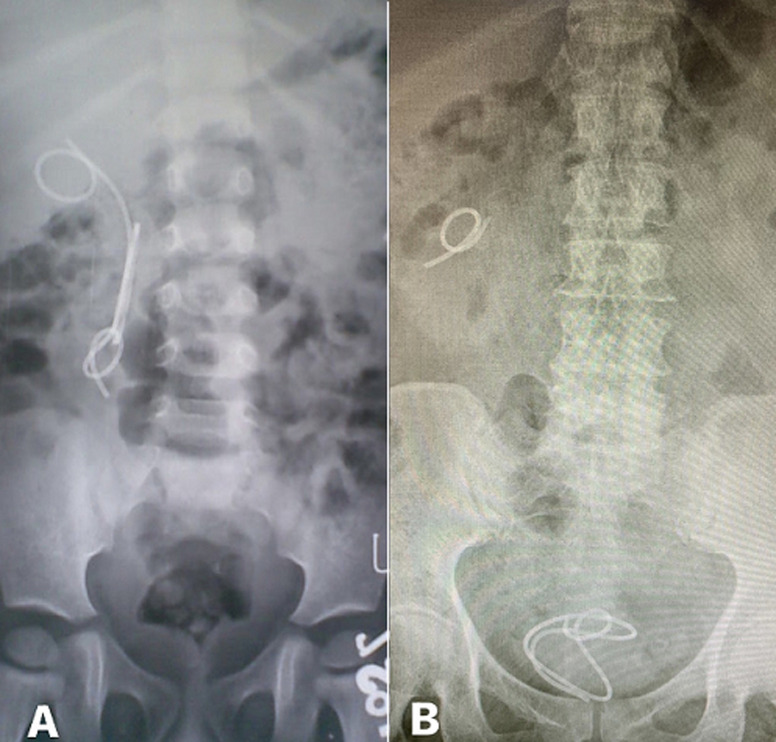
A) plain radiograph showing proximal migration of a retained ureteral stent; the stent was removed using ureterorenoscope; B) a plain radiograph showing fragmented retained ureteral stent with migration of lower end into the bladder which was removed cystoscopically; the proximal end was removed using a ureterorenoscope

**Figure 3:**
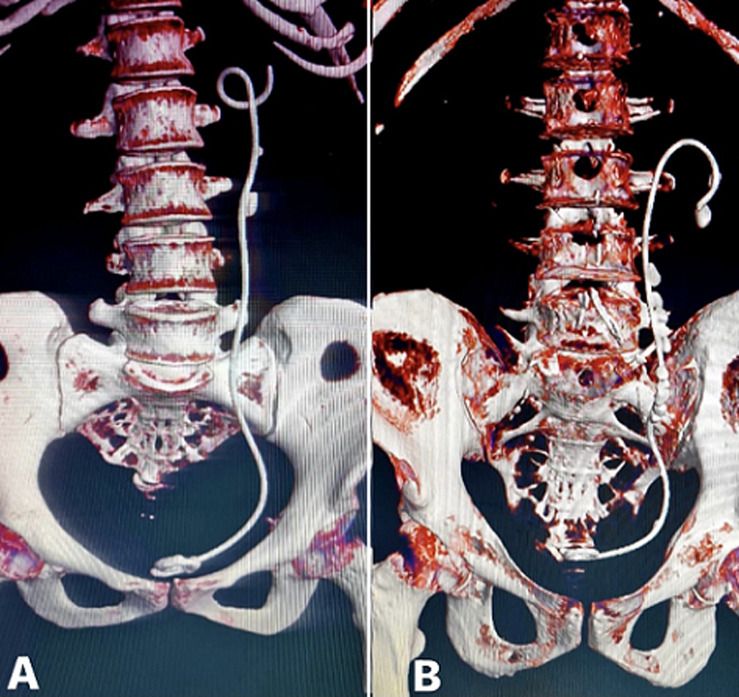
A) 3-dimensional reconstructed computed tomography images showing retained ureteral stents with encrustation of the lower coil and stone formation along the body which needed cystolithotrity and ureteroscopic lithotripsy; B) 3-dimensional reconstructed computed tomography images showing retained ureteral stents with encrustation and stone formation along the upper coil and the body which needed percutaneous nephrolithotomy and ureteroscopic lithotripsy

**Methodology:** the preoperative laboratory evaluation in such patients consisted of urinalysis, urine culture and antibiotic sensitivity, serum creatine level, and whole blood count. Patients underwent radiological assessment in the form of ultrasonography, X-ray, NCCT, or CECT of the KUB region to assess the anatomy, stone burden, and stent condition (Figure 1(A, B), Figure 2(A, B), Figure 3(A, B)). Patients whose kidney´s functional status was doubtful based on their radiological and laboratory investigations, were subjected to a functional nuclear scan in form of diethylenetriamine penta-acetic acid (DTPA) or ethylene di-cysteine (EC) scan. Various endourological procedures (single/multistage) were used to render the patients stent free and clear the associated stone burden. Patients with clinical and radiological features suggestive of pyonephrosis underwent USG-guided emergency percutaneous nephrostomy (PCN) insertion followed by definitive management for their retained stents after stabilisation. Procedures that were performed were cystolithotripsy (CLT), percutaneous cystolithotrity (PCCL), ureteroscopic lithotripsy (URSL), percutaneous nephrolithotomy (PCNL), extracorporeal shockwave lithotripsy (ESWL), simple endoscopic stent removal, open pyelo/nephrolithotomy, and simple nephrectomy.

**Figure 4:**
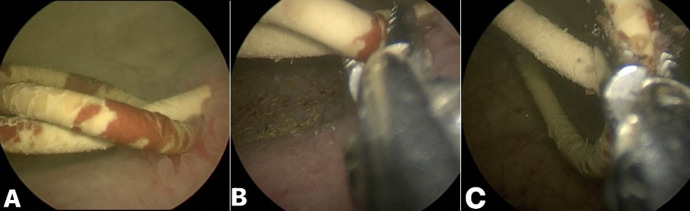
A) cystoscopic view of an encrusted retained ureteral stent with knotting of the lower coil: B,C) cystoscopic view showing lower coil stent encrustation being broken using grasping forceps which helped in unknotting of the lower coil

**Figure 5:**
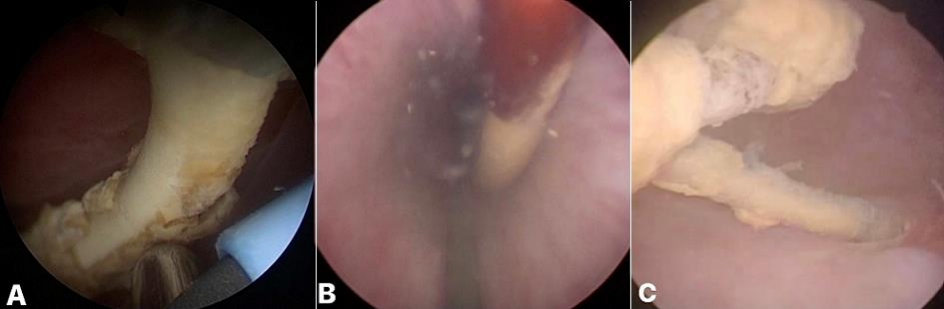
A) endoscopic view of percutaneous nephrolithotomy done for retained ureteral stent with upper coil encrustation; B) endoscopic view of ureteroscopic lithotripsy for retained ureteral stent with encrustation involving body of the stent; C) endoscopic view of retained ureteral stent with lower coil encrustation which needed cystolithotrity

All patients received pre and post-operative urine culture-specific antibiotics prophylaxis. In cases where minimal stent encrustation was present, a gentle attempt was made to remove the stent with the help of grasping forceps under fluoroscopic guidance (Figure 4 (A, B, C)). If resistance was encountered or the stent failed to uncoil, procedures were abandoned, and ancillary procedures like URSL or PCNL were performed (Figure 5 (A, B)). In all cases, a pneumatic lithotripter was used as an intracorporeal lithotripter. Extracorporeal shockwave lithotripsy was tried only for the patients with upper-coil encrustation/associated stone size of less than 1 cm and Hounsfield unit < 1000, otherwise PCNL was done using an 18 or 22 Fr rigid nephroscope. For encrustation at the lower coil of the stent, fragmentation was done using transurethral CLT or PCCL (Figure 5C) For encrustations involving the stent body, URSL was done using 6/7.5 Fr semirigid ureteroscopes. A new DJ stent was placed in patients who underwent URSL, PCNL, or pyelolithotomy. In all cases, these new stents were removed four to six weeks post-operatively. Before stent removal X-ray/USG/NCCT KUB confirmed the stone-free status on case to case basis.

At our institute until 2015, to ensure timely follow-up in patients with indwelling stents, we used to counsel the patient regarding the indwelling stent and give an appointment at the time of discharge for stent removal/change. Such information was printed on the discharge cards handed over to the patient. However, from 2015 onwards, we incorporated the “three steps” prevention check method where patients and their relatives are made aware of the indwelling stents (showing post-operative X-ray). Secondly, we started stamping the discharge cards with the required information related to indwelling ureteral stents in patients´ language. Thirdly, their addresses and phone numbers are recorded in the “stent diary”. A first reminder call is given four weeks after the discharge. The second reminder call is eight weeks and the third reminder call is twelve weeks after the discharge. The information regarding the same is also sent via text messages.

**Data processing and analysis:** at the end of the study, the data was compiled and comprehended. Statistical analysis was performed using Microsoft excel 2010 and IBM SPSS Statistics for Windows, Version 22.0 (IBM Corp., Armonk, NY, USA). Descriptive statistics were applied, and the data was interpreted. The categorical variables were presented as absolute frequencies with their respective percentages (%), whereas continuous variables were presented as mean and range.

## Results

We retrospectively analysed the data of 114 patients (51 males and 63 females) between January 2010 and January 2020 who were managed for retained ureteral stents. The patient demographics, indications for primary stenting, site of stent encrustation, indwelling time, and the primary reason for retained stent are shown in Table 1. Of the 114 patients analysed, 82 patients (71.9%) were operated on at an outside centre, and 32 patients (28.1%) were our follow-up patients. The mean indwelling time of stents was 16.11 months, with a range of 7 to 98 months. Most of them had presented with abdominal pain (62 patients, 54.4%), dysuria (41 patients, 35.1%), haematuria (37 patients, 32.5%), and a history of frequent UTIs (21 patients, 18.4%). However, in 24 patients (21.1%), stents were detected incidentally, who were asymptomatic. Six patients (5.3%) presented with features suggestive of pyonephrosis.

**Table 1:** patient demographics and ureteral stent characteristics

Variables	Number	Percentage (%)
Number of patients	114	-
Mean age (years)		
**Gender**	38.4 (9-66)	-
Male	51	44.7
Female		
**Indication for primary stent**	63	55.3
Ureteric stone surgery	58	50.9
Renal stone surgery	39	34.2
Ureteric stricture	11	9.6
Obstructive uropathy due to malignancy	4	3.5
Pyeloplasty		
**Location of stent encrustation**	2	1.8
Upper coil	45	39.5
Body	45	39.5
Lower coil	58	50.8
More than one part	45	39.5
None		
**Indwelling time**	21	18.4
Mean indwelling time (months)		
**Primary reason cited by patient for retained stent**	16.11 (7-98)	-
Poor compliance	52	45.6
Unaware	40	35.1
Misconception (lifetime)	14	12.3
Misconception (dissolve)	8	7.0

**Figure 6:**
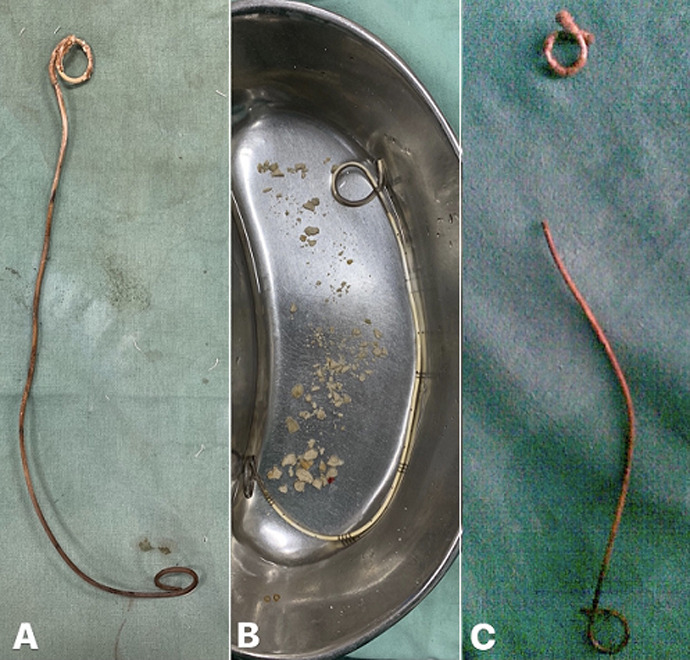
A) retained ureteral stent with upper coil encrustation removed via percutaneous nephrolithotomy; B) retained ureteral stent with lower coil encrustation removed after cystolithotrity; C) fragmented retained ureteral stent with encrusted upper coil removed via percutaneous nephrolithotomy and rest of the stent via cystoscopy

An average of 1.7 sessions (range 1-4) were needed to make the patients´ stent free. During these sessions, a single or multiple procedures (endoscopic/ open/ combined) were performed to facilitate the removal of the retained stent and associated stones (Table 2). There were 21 patients in whom no part of the stent was encrusted. They all underwent successful stent removal without the need for any ancillary procedure. In 23 patients (20.2%), transurethral CLT was done, and 2 patients (1.7%) with large lower coil encrustation needed PCCL. Patients with associated encrustation in the upper coil or body needed additional procedures in the form of PCNL, URSL, or both (Figure 5 (A, B, C)). There were four patients (3.5%) in whom all three parts of the stent were encrusted. They were managed successfully using three endoscopic procedures CLT, PCNL, and URSL, in single or multiple sessions. Spontaneous stent migration (up/down) from the normal position was encountered in 16 patients (14.0%), and spontaneous stent fragmentation was seen in 15 patients (13.2%) (Figure 2 (A, B)). For patients who had stent fragmentation, all parts were retrieved via endoscopic procedures by using cystoscopy or URS or PCNL, or combined approaches (Figure 6 (A, B, C)). Extracorporeal shockwave lithotripsy was successfully performed in 10 patients (8.8%). However, more than one ESWL session was needed. There were two patients (1.8%) who underwent open pyelolithotomy for upper coil encrustations with a large stone burden in the renal pelvis (size > 3 cm).

**Table 2:** surgical procedures performed to remove retained ureteral stents

Surgery performed	Number	Percentage (%)
Simple endoscopic stent removal	21	18.4
PCNL	2	1.8
URSL	12	10.5
CLT	23	20.2
PCCL	2	1.7
ESWL	6	5.3
PCNL+URSL	8	7.0
PCNL+CLT	10	8.8
URSL+CLT	11	9.6
ESWL+CLT	2	1.8
ESWL+URSL	2	1.8
PCNL+URSL+CLT	4	3.5
Open nephrectomy	3	2.6
CLT + open nephrectomy	6	5.3
Open pyelolithotomy	2	1.8

Six patients with pyonephrosis at admission underwent PCN insertion followed by definitive procedures. CLT: cystolithotripsy; ESWL: extracorporeal shockwave lithotripsy; PCCL: percutaneous cystolithotrity; PCN: percutaneous; PCNL: percutaneous nephrolithotomy; URSL: ureteroscopic lithotripsy

These patients insisted and opted for open surgery over PCNL to remove the encrusted stents and associated stones. Out of 114 patients, 51 patients (44.7%) needed re-stenting. These were the patients who underwent either PCNL/URSL (49 patients) or open pyelolithotomy (two patients). Before the removal of the re-inserted DJ stent, all these 51 patients underwent NCCT KUB. None of the patients post URSL or open pyelolithotomy had any residual stone/stent fragment. However, five of the 24 patients (20.8%) post PCNL had residual stone fragments. Second look nephroscopy was not required in any of these patients as the residual fragments on NCCT KUB were less than 4 mm in size. The most common post-operative complication was urosepsis (18 patients, 15.8%). Blood transfusion was needed in four patients (3.5%), and three patients (2.6%) had grade 1 ureteric injury. Out of 114 patients, nine patients (7.9%) had permanent loss of renal unit function. They all underwent open nephrectomy. Out of these nine patients, six had lower coil encrustation, which was managed by performing CLT before open nephrectomy. The majority (66 patients, 57.9%) of the ureteral stents retrieved were 6Fr multilength polyurethane DJ stents.

At our centre from January 2010 to January 2015, a total of 1864 patients underwent DJ stent placement for various reasons, out of which 21 patients (1.1%) had retained stents on follow-up. From 2015 to 2020, a total of 2007 patients underwent DJ stent placement, of which 11 patients (0.5%) had retained stents on follow-up. These 32 patients were included in the study. Poor compliance on the part of the patient (45.6%), unawareness (35.1%), and misconception that the stent would last a lifetime (12.3%), were the most common reasons for retained DJ stents. The incidence rate of retained stents fell from 1.1% to 0.5% after we started using the “three steps” prevention check method to ensure timely follow-up of the patients.

## Discussion

The use of silicone ureteral splints to relieve the ureteral obstruction was first reported in 1967 [7]. Although ureteral stents play an essential and integral part in managing various urological conditions, they are to be removed or changed on a timely basis. When these stents are left indwelling for a prolonged time it results in various complications, including migration, fragmentation, and stone formation [2,4]. The incidence of encrustation is directly related to the duration of indwelling time [1,2]. El-Faqih *et al*. [8], found that encrustation increased from 9.2% at < 6 weeks to 47.5% at six to 12 weeks to 76.3% at > 12 weeks of indwelling time. In our study, the mean stent indwelling time was around 16 months (64 weeks). Therefore, the DJ stent needs to be replaced or removed within six weeks to six months [5,8]. Apart from prolonged indwelling time (which is the most crucial factor for encrustation), the other reasons for encrustation are stone disease, urinary sepsis, chemotherapy, pregnancy, chronic renal failure, and metabolic or congenital abnormalities [7,9]. In our study, most patients (85.1%) had a prior history of stone disease, which is a risk factor for stent encrustation. Other factors which likely played a role in stent encrustation were prolonged indwelling time and UTIs.

The mechanism of encrustation appears to be dependent on several factors. Stent encrustation can be found in sterile as well as infected urine [4]. Urease-producing organisms in urine hydrolyse urea to produce ammonia, which causes alkalization of urine and favours magnesium and calcium precipitation as struvite and hydroxyapatite onto the bacterial biofilm layer on the stent surface [4,9]. Other factors include properties of the stent biomaterial and metabolic abnormalities similar to those seen in urolithiasis, such as hypercalciuria, hyperoxaluria, hypocitraturia, homocystinuria, and hyperuricosuria [9]. Encrustation results in a reduction in peri-stent and intraluminal flow, which gradually worsens over time as the burden of encrustation increases with prolonged indwelling time. This results in stent failure, urinary tract obstruction, UTIs, and impaired renal function [6].

Presentation of patients with forgotten DJ stents may vary. We found abdominal pain (54.4%), dysuria (35.1%), and haematuria (37 patients, 32.5%) as the most common presenting symptoms. In a study by Damiano *et al*. [10], flank pain (25.3%) and storage lower urinary tract symptoms (18.8%) were the most common. The forgotten stent may be asymptomatic and “remembered” only when incidentally detected [1]. In our study, 24 patients (21.1%) had retained stents that were detected incidentally. These patients were asymptomatic and were referred to us from another specialty after stents were detected on radiological investigations done for other reasons. Patients with forgotten stents should be evaluated by radiological investigations in the form of NCCT/CECT KUB [5]. All our patients underwent preoperative NCCT/CECT KUB to assess the site of stent encrustation/stone burden and to decide the treatment strategy. Endoscopic management is successful in most cases, but a multimodal approach in single or multiple sessions might be needed [6]. Although we used more than six months of indwelling time to define retained/forgotten stents, even a shorter period of indwelling time can cause difficulty in removing ureteral stents. The need for an additional stent removal procedure is likely if indwelling time exceeds three months [11]. Bultitude *et al*. [12], reported difficulty during stent removal via cystoscopy in 42.8% of patients within four months and 14.3% at two months. Okuda *et al*. [13], reported 15 irremovable ureteral stents in Japanese patients with mean indwelling times of 20 months. Bukkapatnam *et al*. [14], described one-step removal of encrusted, retained ureteral stents, whereas Mohan-Pillai *et al*. [15], mentioned an average of 2.5 endourologic approaches to achieve stent-free status. In our study, on average, 1.7 sessions were needed per patient to clear the stent and associated stone burden. During these sessions, single or multiple procedures were performed.

In our study, several endourological approaches and open pyelolithotomy were needed depending on the location of encrustations, and stent location (migration/fragmentation), and associated stone burden. Clearance rates ranging from 75% to 100% have been obtained by others using a combination of ESWL, URSL, PCNL, and percutaneous nephrostomy plus chemolysis with Suby´s Solution G [4,5,11,14]. Never, significant force should be used to attempt stent removal, as severe ureteral injury or stent fragmentation may happen [16,17]. It is better to perform an ancillary procedure rather than converting a situation from bad to worse. Open procedures may be needed in case of failure of the endourological procedure, large stone burden requiring multiple punctures on PCNL, or patient´s preference towards open surgery [9,17]. Post-operative imaging in the form of X-ray/NCCT KUB is ideal for documenting any residual stones [17,18].

The intra and post-operative complications are minimal when it comes to the operative management of retained ureteral stents [3,4,9]. In our experience, the most common post-operative complications were urosepsis (15.8%), need for blood transfusion (3.5%), and grade 1 ureteric injury (2.6%). Also, prolonged obstructed encrusted stents can cause permanent renal unit loss, as was seen in nine of our patients (7.9%) who needed a nephrectomy. In a poorly functioning, non-salvageable kidney with a significant stone burden and encrusted stent, nephrectomy should be considered [16,17]. The best treatment for the management of forgotten indwelling stents remains prevention [3]. They cause physical, psychological, and financial stress to the patient. In some cases, it can even lead to life-altering consequences resulting from irreversible loss of renal unit function. It is unjust to our patients that they must suffer this morbidity when in most cases, this situation can be avoided altogether by ensuring proper counselling.

Patient unawareness remains one of the most common reasons behind forgotten stents [18]. In our study, the most common reasons behind failure to follow-up timely were poor compliance on the part of the patient (45.6%), unawareness (35.1%), and misconception that the stent would last a lifetime (12.3%). Making the patients aware of an indwelling foreign body and when to follow-up is the key to avoiding the problems associated with retained stents. Various efforts have been made on this front in the form of stent registry, smartphone applications, and web-based systems sending timely reminders to the patients and doctors [2,4,18]. Since we started using the “three steps” prevention check method to ensure timely follow-up of the patients, the incidence rate of retained stents fell from 1.1% to 0.5%. Unfortunately, despite many reminders, some patients still failed to follow-up timely.

**Study limitations:** as a retrospective study design, it is subject to inherent limitations. There is bound to be selection and recall bias. The majority of the patients (71.9%) were referred from outside hospitals, so the complete details of primary surgery were lacking. Stone analysis was not done in the majority of our patients. No objective method was used pre-operatively to measure the burden of stent encrustation. In our study, we did not use a flexible ureteroscope and laser lithotripsy, which could have provided better stone clearance rates.

## Conclusion

Retained ureteral stents continue to pose a challenge in the field of urology. They are a significant source of morbidity, which is avoidable by ensuring their timely removal. Sincere efforts should be made to make patients aware of an indwelling foreign body. Although stents can be successfully removed by using the multi-modal approach, prevention is the best strategy.

### What is known about this topic

- Retained ureteral stents continue to be a source of significant morbidity in current urological practice;
- There are several factors that lead to the prolonged indwelling time of ureteral stents, mainly poor compliance on the patient’s part and unawareness.

### What this study adds

- Endourological procedures to remove such stents are successful in the majority of the cases, however, single or multiple sessions might be needed;
- Prevention is the key, which entails ensuring timely follow-up of the patients with indwelling stents;
- The “three steps” prevention check method helps in reducing the incidence rate of retained stents.
